# Astral Projection: A Strange Out-of-Body Experience in Dissociative Disorder

**DOI:** 10.7759/cureus.17037

**Published:** 2021-08-09

**Authors:** Varchasvi Mudgal, Rashmi Dhakad, Rahul Mathur, Ujwal Sardesai, Virendra Pal

**Affiliations:** 1 Psychiatry, Mahatma Gandhi Memorial Medical College, Indore, IND

**Keywords:** astral projection, out of body experience, dissociative disorder, dissociative fugue, dissociative identity disorder

## Abstract

Out-of-body experiences (OBEs) are hallucinatory visual experiences that involve seeing the physical body placed in an external visual space. Many psychiatric disorders, brain dysfunctions, pharmacological agents, and altered psychological states are reportedly associated with these phenomena. OBEs have been linked to various brain lesions, particularly in the parietal and temporal regions, psychiatric disorders, severe emotional states like a near-death experience, substance use, migraine, and epilepsy, but very few have been reported in dissociative identity disorder. In this report, we present the case of a 15-year-old male patient who described a strange experience where he found himself to be floating outside his own body while he visualized his own body from a third-person perspective. On further evaluation, a diagnosis of dissociative identity disorder and dissociative fugue was formulated. The patient showed improvement after undergoing abreaction, hypnosis, and relaxation training along with supportive psychotherapy. Dissociative disorders occur due to an internal conflict between ego and self, when a person is unable to successfully repress a traumatic experience, or when a repressed memory or experience comes out of the cocooned barrier, leading to an altered state of perception and self-experience, which is described by the patient as OBE. This report presents a scarce differential in the context of psychiatric illness, which might be helpful in the formulation of approaches toward management in cases of such OBE, making it a strange yet intriguing addition to the literature.

## Introduction

Out-of-body experiences (OBEs) are visual hallucinatory experiences in which the individual sees his/her own physical body placed in external visual space, like a reflection in the mirror. Many psychiatric disorders, altered psychological states, brain dysfunction, psychoactive substances, and pharmacological agents are reportedly associated with these phenomena [1]. There are about six major variants of the autoscopic phenomenon, namely negative autoscopy, which refers to the failure to perceive one’s own body either in a mirror or when looked at directly; inner autoscopy, which pertains to the experience of visual hallucinations of internal organs in extra-corporeal space looked at directly; the feeling of presence where the patient has a distinct feeling of the physical presence of another person; heautoscopy proper where the patient does not localize himself in the position of the mirror image; autoscopic hallucination, which refers to the patient seeing an exact mirror image of himself, or of his face or trunk; and OBE, which is characterized by the projection of an observing (psychological) self in extrapersonal space seemingly totally dissociated from the physical body [2-3]. 

In OBE, the projection of an observing (psychological) self is seen in extra-personal space that seems totally dissociated from the physical body. In this phenomenon, the patient from a location distinct from his physical body sees himself and the surrounding world. These phenomenological states are divided into three categories, namely disembodiment, the sense of seeing the body from a remote and raised visuospatial perspective (extra-corporeal egocentric perspective), and the sense of seeing one’s own body from an aerial position, which is sometimes referred to as astral projection [3-4]. OBEs have been linked to various brain lesions, particularly in the parietal and temporal regions, psychiatric disorders, severe emotional states like a near-death experience, substance use, migraine, and epilepsy, but very few have been reported in dissociative identity disorder. The recent Marvel movie “Dr. Strange”, and "The Last Hour", an over-the-top (OTT) series, have dealt with this fascinating phenomenon where the character experiences a form of depersonalization with his parasomatic component coming out of his body and is visualized by his own self from a third-person perspective described as astral projection. An OBE can be understood as a form of dissociation and transpersonal experience. There is a plethora of anecdotal but little empirical evidence related to the connection of dissociation and OBE, implying a shortcoming in the understanding of OBEs [5-6]. This strange and rare phenomenon has usually been reported in organic states, under substance influence, and intense emotional states. In this report, we present a case involving a lucid state of OBE in the context of dissociative identity disorder.

## Case presentation

The patient was a 15-year-old male child belonging to an urban, middle socioeconomic class, who was living with his father; his mother had abandoned him as a child. He presented to the department of psychiatry with his father, who reported that the child had frequently run away from home in the past three to four years; he had started behaving differently and had shown decreased social interest, irritability, and persistent sadness of mood for the past two to three months. The father reported that after the patients' most recent disappearance from home, he had been contacted by police officials of another state one month after the patient had run away; they had informed him that the patient was at a childcare facility and could be picked up from there. The patient described that after he had run away, he would assume the identity of an 18-year-old Mr. S, who was an electrician. During subsequent interviews, patients described an unusual experience where he had found himself to be floating outside his own body while he visualized his own body from a third-person perspective. This incident had occurred during one of his fugue states in another city; he described being inside a hospital room with doctors who were questioning him about his current state. Later, he had felt like someone else had occupied his body and his soul had left his body and floated up to the ceiling and was completely detached from his body; from his visuospatial angle, he had been able to visualize his own body, which had been very clear while the parasomatic body had not been well defined and he could only see its hands, He had tried tirelessly to reach back to his original self but had been unable to do so. He had seen his body being interviewed by the doctors to whom his parasomatic image tried to reach out, but he had little control over its movement and kept on floating. This episode had only lasted for a short period of time, about 10-15 minutes as estimated by the patient, during which he had remained in the air observing his original self and in very little control of the parasomatic body. The original self had been replying briefly to the interviewers as per the patient as he described it was not him who was in control of his original self.

The patient’s past history revealed that he had fled from his residence on three occasions previously, but he did not remember the reason for fleeing. His medical history was negative for symptoms of epilepsy, migraine, syncope, cerebrovascular accident, neurological deficit, etc. There was no history of episodes of hyperpyrexia warranting admission. No psychiatric illness or substance dependence was present in the family. His mother had left home when he had been a year old. His upbringing was done by his father. He was living in a joint family and as per the patient, the relations between family members were not congenial. Also, as per the patient, his father was very aggressive and short-tempered, and frequently hit him brutally, and he had sustained multiple injuries as well. This was why he frequently ran away, according to the patient.

Personal history revealed that the patient had been a full-term normal delivery with appropriate developmental milestones. The patient had speech disorder in the form of lisping since childhood and had traits conforming to conduct disorder, such as bullying young children and threatening them, behaving deceitfully, lying, and manipulating people to obtain favors, episodes of truancy from school, staying outside beyond home curfew, etc. He had a prior relationship with three girls, of which his father had not approved, and those had been short-lasting. He had fallen foul of the law during his time away from home, and he had spent few months in a correctional facility for juveniles in Gujarat, India. After corroboration from reliable informants and patient interviews, substance use was ruled out; also, the patient did not display any features of substance withdrawal during his inpatient stay. Premorbid personality assessment revealed that he was an introvert, optimistic regarding new situations, short-tempered, and self-dependent.

Vitals including temperature were unremarkable, ruling out hyperpyrexia. No other abnormality was detected during general and systemic examinations; an otorhinolaryngology opinion was also sought to rule out vestibular defects, which could contribute to OBEs. During the serial mental status examination, a rapport was built with the patient and he revealed that the lack of a mother and harsh parenting by his father had led to him to a state of persistent stress and he wished that this fugue-like state would end and described a feeling of helplessness and persistent sadness, which was also evident in his affect. He did not have any delusions or hallucinations and denied any change in sense of agency routinely. His speech was appropriate with a slight lisp, and his psychomotor activity was normal. Cognitive tests were unremarkable and appropriate to age. Baseline investigations including hemogram, liver, and renal function tests were within normal limits. Electroencephalography (EEG) did not reveal any abnormality. CT scan of the brain did not show any pathological findings.

A diagnosis of dissociative identity disorder and dissociative fugue was formulated along with secondary depression as per the International Classification of Diseases, Tenth Revision (ICD-10). His Adolescent Dissociative Experiences Scale-II (A-DES) score was 118/300 (suggestive of moderate dissociative experience) on initial assessment. Kutcher Adolescent Depression Scale (KADS) was used to assess depressive symptoms; the patient scored 9, which suggested possible depression. The neuropsychological assessment involved IQ assessment, and the Rorschach test was suggestive of depressive and anxiety disorder but no psychotic features. As a therapeutic modality, abreaction was performed using a guided interview along with injecting 1 mg Intravenous lorazepam after obtaining written consent from the patient and his guardian. He had another OBE during his interview, which was similar to the previous one. During the interview, he slipped into a trance-like state and gave brief answers. He stated as follows: “below me, I saw my body, from outside lying on bed and the doctor standing near me was asking some questions.” His voice was changed, his lisping was absent, and his tone was loud, and within few seconds, he started shouting but later calmed down within a few minutes. After the abreaction, his stress symptoms and depression improved. Escitalopram was initiated at a dose of 10 mg, which was titrated up to 15 mg in four weeks along with clonazepam 0.25 twice a day. Abreaction, hypnosis, and relaxation training along with supportive psychotherapy were provided to the patient in a structured format. His father was psycho-educated about his illness and was briefed about interpersonal conflict management. After four weeks of inpatient management, the symptoms of dissociation and the OBE phenomenon resolved. The patient was followed up for the next six months and did not report any further dissociative state or OBE.

## Discussion

OBEs are perceptual disturbances where the subjectively experienced location of the perceiving self is altered. It is characterized by a unique sense of visuospatial orientation and the presence of a real and parasomatic component. Dissociative experiences are very unusual and can present in different forms, which in turn can lead to reality-altering perceptions in the patient. The etiopathogenesis behind them could be psychopathological, neurological, social, or environmental, and secondary to their cultural relevance and acceptance, they could be presented in clinical settings and at times may remain undetected. One of the variants of dissociative experiences is the OBE. OBE can be understood as a state of depersonalization/derealization that manifests as feeling disconnected from one’s body/surroundings, unusual body experiences, with a background of emotional blunting. The literature on the cause of OBE mainly entails various neurological conditions like epileptic seizures and migraine, deficient visual, vestibular, and multisensory processing, near-death experiences, and psychedelic drug use. These peculiar experiences have been described as secondary to the psychopathology of psychiatric disorders such as schizophrenia, personality disorders, depersonalization, anxiety, dissociative disorders, and depression [7-8].

Delving into the psychopathological angle of OBE, we found increasing support for the theory that attributes the origins of OBEs to the mismatch and deficient working of neurocognitive processes that maintain self-awareness, which in turn creates distortions in the sense of agency, presence, and embodiment. The malfunctioning of multisensory integration can lead a person to transcend into a state of dissociation and associated perceptual distortions, which are construed as strange projections of self out of the body. Dissociative disorders represent a tussle between conscious and unconscious, when a person is unable to successfully repress a traumatic experience, or when a repressed memory or experience comes out of the cocooned barrier, leading to an altered state of perception and self-experience, which is described by the patient as OBE [9-10]. In our patient, his mother’s absence since childhood may have hindered regular feeling of attachment, evidenced by the lack of his bonding with his primary caregiver and association only with a small social group, which could have predisposed him to the dissociative state.

## Conclusions

In our report, OBE was found secondary to dissociative disorder, which improved with the help of psychotherapy and psychotropics. The various differentials were ruled out using various investigations, vestibular defects testing, neuroimaging, and neuropsychological tests. Given the patient's response to psychopharmacology and the absence of further OBE in him, there is ample evidence to suggest that astral projection can be construed as a part of the dissociative experience rather than a breakdown of the sensory neural system or any significant organic state in the current report. Thus, this report presents a scarce differential in the context of psychiatric illness, which might assist in the formulation of approaches toward management in the case of such OBEs, making it a strange yet intriguing addition to the existing body of literature.
